# Development of a Lentiviral Reporter System for In Vitro Reprogramming of Astrocytes to Neuronal Precursors

**DOI:** 10.3390/biology14070817

**Published:** 2025-07-05

**Authors:** Anna Schnaubelt, Guoli Zheng, Maryam Hatami, Johannes Tödt, Hao Wang, Thomas Skutella, Andreas Unterberg, Klaus Zweckberger, Alexander Younsi

**Affiliations:** 1 Department of Neurosurgery, Heidelberg University Hospital, 69120 Heidelberg, Germany; schnaubelt.anna@web.de (A.S.); guo3li4.zheng4@gmail.com (G.Z.); hallking0124@gmail.com (H.W.); andreas.unterberg@med.uni-heidelberg.de (A.U.); k.zweckberger@skbs.de (K.Z.); 2 Medical Faculty, University of Heidelberg, 69120 Heidelberg, Germany; setazeshmersana2015@gmail.com (M.H.); thomas.skutella@uni-heidelberg.de (T.S.); 3 Department of Neuroanatomy, Institute for Anatomy and Cell Biology, University of Heidelberg, 69120 Heidelberg, Germany

**Keywords:** astrocyte reprogramming, neuron conversion, neuronal precursor cells, Oct4, Sox2, Klf4, neuroregeneration, lentiviral vectors, brain repair

## Abstract

Astrocytes, which proliferate after brain injury, represent a promising target for cellular reprogramming due to their abundance and ability to support brain repair. In this study, we investigated the in vitro reprogramming of primary cortical astrocytes from neonatal rats into neuronal precursor cells (NPCs) using the transcription factors Oct4, Sox2, and Klf4 (OSK), delivered via lentiviral vectors. We designed a reporter system to trace the conversion of astrocytes to NPCs and neurons by using GFAP-driven iCre and Nestin- or Synapsin1-driven fluorescent reporters. After transduction, we observed morphological changes and the expression of neuronal markers in some cells, while many cells remained in a transitional state, expressing both astrocytic and neuronal features. Importantly, the study was not designed to quantify reprogramming efficiency or demonstrate full astrocyte-to-neuron conversion but rather to establish and evaluate a traceable reporter system. Our data suggest that OSK-mediated reprogramming in this in vitro model can initiate conversion of astrocytes to neuronal precursor-like cells, although the process is complex and incomplete within the one-week timeframe. We also highlight limitations in co-transduction efficiency and potential silencing of the reporter system during reprogramming. These findings provide an initial technical platform to explore astrocyte reprogramming in vitro and inform future studies aiming to refine these methods and apply them in vivo.

## Simple Summary

Astrocytes are a major cell type in the brain that can potentially be reprogrammed to become neuronal precursor cells (NPCs), offering insights for future regenerative therapies. In this study, we developed an in vitro approach to reprogram rat cortical astrocytes using the transcription factors Oct4, Sox2, and Klf4 (OSK), delivered via lentiviral vectors. We also established a reporter system to monitor the transition from astrocytes to NPCs. Our experiments showed that while some astrocytes began to express neuronal markers, many cells remained in a transitional state, reflecting the complexity of the reprogramming process. These findings provide a technical foundation for further studies exploring astrocyte reprogramming in vitro and in vivo.

## 1. Introduction

Traumatic brain injury (TBI) is a leading cause of long-term disability and cognitive impairment worldwide, affecting millions of individuals annually and posing a major public health challenge. Despite extensive research, effective treatments that can restore lost neuronal function or promote regeneration remain limited [1,2,3]. A key obstacle in brain repair lies in the limited regenerative capacity of the central nervous system (CNS) [4,5,6]. In this context, cellular reprogramming has emerged as a promising approach to generate new neurons or neuronal precursors, offering potential avenues for regenerative strategies. Following the pioneering work of Takahashi and Yamanaka, who reprogrammed somatic cells into induced pluripotent stem cells (iPSCs) using the transcription factors Oct4, Sox2, Klf4, and c-Myc [7,8], various strategies have explored direct or indirect reprogramming of glial cells into neuronal phenotypes [9,10,11,12].

Astrocytes are particularly attractive targets for such strategies, as they are abundant, become reactive after CNS injury, and contribute to glial scar formation [13,14,15,16,17]. Moreover, their proliferative response and transcriptional plasticity provide an accessible entry point for reprogramming interventions. Previous studies have shown that astrocytes can be reprogrammed into neuronal precursor cells (NPCs) or neurons by overexpression of key transcription factors [9,10,11,12]. However, the processes underlying such conversion are complex, often incomplete, and influenced by factors including cell heterogeneity, culture conditions, and the delivery methods of reprogramming factors [11,18,19].

In this context, we aimed to establish a modular and traceable in vitro system to assess astrocyte-to-neuron or astrocyte-to-NPC conversion. Using a lentiviral delivery system, we overexpressed the OSK trio (Oct4, Sox2, Klf4), which has previously been shown to promote neurogenic plasticity in various settings. In parallel, we developed a GFAP-promoter-driven reporter system to visualize astrocyte identity and monitor lineage transitions toward Nestin^+^ NPCs or Synapsin-1^+^ neurons [20,21].

While our study does not model TBI directly, it addresses key methodological questions that are highly relevant for future translational research in neurotrauma. Specifically, we evaluate the feasibility and limitations of using OSK-mediated reprogramming in primary astrocyte cultures, with a focus on marker dynamics, transduction efficiency, and the performance of a multicomponent reporter system. This technical groundwork may help optimize future in vivo studies that aim to convert reactive astrocytes into neurons in the injured brain.

## 2. Materials and Methods

### 2.1. Isolation and Culture of Primary Astrocytes

Primary astrocytes were isolated from the cortex of postnatal day 1–2 (P1–P2) Wistar rat pups, in accordance with protocols approved by the Animal Care Committee of the Federal Government of Baden-Württemberg, Germany (T58-19, T-31/20, T-05/22). We used neonatal rats for their higher astrocyte proliferative potential, which facilitates in vitro culture, though this choice represents a limitation for direct translation to adult injury models.

Briefly, animals were anesthetized with isoflurane, and their necks were disinfected with 70% ethanol to prevent contamination. Decapitation was performed with sharp scissors, and the heads were placed on sterile gauze. Brains were carefully dissected under a stereomicroscope and transferred to 10 cm Petri dishes containing ice-cold phosphate-buffered saline (PBS, Gibco, Carlsbad, CA, USA). Meninges were removed using fine forceps. The cortices were then transferred to new dishes with ice-cold PBS and minced into small pieces to improve dissociation.

Tissue fragments from 3–4 pups were pooled into 15 mL Falcon tubes containing 5 mL of digestion solution (0.05% trypsin/EDTA (Gibco, Carlsbad, CA, USA) and 0.1 mg/mL DNase I (Roche, Basel, Switzerland) and incubated at 37 °C for 15 min, with gentle manual shaking every 5 min. Digestion was stopped by adding 7 mL of astrocyte culture medium (DMEM + 4.5 g/L glucose, 10% FBS, 1% penicillin/streptomycin; see also Table 1). The tissue was dissociated by repeated pipetting (20–30 times with a 10 mL pipette) and centrifuged for 5 min at 1200 rpm. The cell pellet was resuspended in 10 mL astrocyte culture medium, mixed thoroughly (10–15 times with a 10 mL pipette), and filtered sequentially through 70 µm and then 40 µm cell strainers. The filtrate was centrifuged again at 1200 rpm for 5 min, and the resulting pellet was resuspended in 10 mL astrocyte culture medium.

The final cell suspension was plated in poly-D-lysine-coated T75 flasks and incubated at 37 °C with 5% CO_2_. Medium was changed on days 1, 2, 5, and 6 or 7, depending on cell density and growth rate. Microglia and oligodendrocyte progenitor cells were removed by gentle tapping of the flasks during medium changes. Upon reaching approximately 80% confluence (day 6–7), cells were either split into 24-well plates for immunocytochemical characterization or cryopreserved at −80 °C for later use.

### 2.2. Construction of Viral Vectors

To generate the lentiviral transfer vectors for astrocyte reprogramming and reporter-based lineage tracing, we employed the NEBuilder HiFi DNA Assembly method (New England Biolabs, Ipswich, MA, USA). Inserts were amplified by PCR using Q5 high-fidelity DNA polymerase, with primers containing a 20-nucleotide homology region to the cloning vector at the 5′ end and a 20–24-nucleotide sequence specific to the cDNA of interest. All assembled constructs were verified by Sanger sequencing.

The following lentiviral vectors were created:

#### 2.2.1. Tet-O-FUW-OSK (OSK)

Previous studies have shown that using only three transcription factors (Oct4, Sox2, and Klf4; OSK), excluding cMyc, reduces tumorigenic potential compared to the full OSKM combination [22,23,24,25] and that cMyc is not crucial for reprogramming [26,27]. Therefore, we generated the Tet-O-FUW-OSK construct by removing cMyc from the original Tet-O-FUW-OSKM backbone (Addgene plasmid #20321, gift from Rudolf Jaenisch). Specifically, Tet-O-FUW-OSKM was digested with AscI and AfeI (New England Biolabs, Ipswich, MA, USA) to remove the cMyc sequence and part of Klf4. The missing 3′ end of the Klf4 cDNA was PCR-amplified and reassembled into the construct using the following primers:

Klf4-F: 5′-ccctagaggcccatttgagcgctggaccccagctcag-3′;

Klf4-R: 5′-aagcttgatatcgaattcggcgcgcctcaaaagtgcctcttcatgtgtaaggcaag-3′;

#### 2.2.2. pLenti-pSyn1-FLEX-mScarlet-V5 (Syn1-Scarlet Reporter)

This construct serves as an iCre-dependent reporter for mature neurons, driven by the Synapsin1 promoter. We prepared the lentiviral backbone by double digestion of the Tet-O-FUW-Ascl1 plasmid (Addgene plasmid #27150, gift from Marius Wernig) with EcoRI and PspXI. The Synapsin1 promoter was amplified from the pAAV-Syn-jRGECO1a plasmid (gift from A. Agarwal). A V5 epitope tag was synthesized and inserted in frame with mScarlet in the pAAV-CAG-FLEX-mScarlet plasmid (Addgene plasmid #99280, gift from Rylan Larsen). FLEX-mScarlet-V5 was then PCR-amplified and combined with the Synapsin1 promoter to assemble the final construct. The primers used were as follows:

Lenti-Syn-F: 5′-tccagtttggttactcgagtagtgcaagtgggttttaggacc-3′;

FLEX-Syn-R: 5′-gtaccgtcctgcgctctcaggcac-3′;

V5-Top: 5′-[Phos]cgcgcctcacgtagaatcgagaccgaggagagggttagggataggcttacccgaaccctt-3′;

V5-Bottom: 5′-[Phos]gtacaagggttcgggtaagcctatccctaaccctctcctcggtctcgattctacgtgagg-3′;

Syn-FLEX-F: 5′-gagagcgcaggacggtacccttcaggtcg-3′;

Lenti-FLEX-R: 5′-tatcgataagcttgatatcgaattcatcgataagcttgatgatcttcgataacttc-3′;

#### 2.2.3. pLenti-pNestin-FLEX-EmGFP (Nestin-EmGFP Reporter)

This iCre-dependent reporter construct targets neural progenitor cells via the Nestin promoter. The Nestin promoter was amplified from the pNestin-EGFP plasmid (Addgene plasmid #38777, gift from Wei Cui) and combined with FLEX-EmGFP, which was cloned by replacing mScarlet in pAAV-CAG-FLEX-mScarlet with EmGFP. The FLEX-EmGFP cassette was PCR-amplified and assembled to create the final construct. The primers used were as follows:

Lenti-Nest-F: 5′-tccagtttggttactcgagtttgcatgcctgcaggtc-3′;

FLEX-Nest-R: 5′-gggtaccgtccgggtacaattccgcagc-3′;

lox-EmGFP-F: 5′-ttatttggcgcgcctgactttcacttgtacagctcgtccatgcc-3′;

loxp-FP-R: 5′-tacgaagttatcaaggccggcatggtgagcaagggcgag-3′;

FLEX-EmGFP-F: 5′-attgtacccggacggtacccttcaggtc-3′;

Lenti-FLEX-R: 5′-tatcgataagcttgatatcgaattcatcgataagcttgatgatcttcgataacttc-3′;

#### 2.2.4. pGFAP-BFP-T2A-iCre

This construct encodes a bicistronic cassette driven by the GFAP promoter (pGfaABC1D), enabling co-expression of BFP (HA-tagged for detection, provided by J. Sprengel [28]) and iCre recombinase, separated by a T2A self-cleaving peptide. The GFAP promoter was amplified from the AAV-GFAP-NLS-CasRx-NLS-FLAG-U6-DR-SgRNA_Ptbp1-DR plasmid (Addgene plasmid #154001, gift from Hui Yang). The final cassette was assembled in the following order: pGfaABC1D-BFP-HA-T2A-iCre. The primers used were as follows:

Lenti-GFAP-F: 5′-tccagtttggttactcgagtaacatatcctggtgtggagtagg-3′;

BFP-GFAP-R: 5′-ctcgctcatcatggtggcaccggtg-3′;

GFAP-BFP-F: 5′-tgccaccatgatgagcgagctgattaaggagaac-3′;

2A-BFP-R: 5′-ctcgctcatcatggtggcaccggtg-3′;

BFP-2A-F: 5′-gactacgccggatccactagtggctccg-3′;

Lenti-iCre-R: 5′-tatcgataagcttgatatcgaattctcagtccccatcctcgag-3′;

### 2.3. Lentiviral Production

Lentiviral particles were produced using the Addgene protocol with Lenti-X 293T cells (Takara Bio, Kusatsu, Japan). Cells were seeded in T75 flasks at a density of 5.2 × 10^6^ cells/cm^2^ in 15 mL of astrocyte culture medium (without antibiotics) and incubated for 24 h, reaching approximately 80% confluence.

A DNA solution was prepared by combining the lentiviral transfer plasmid (2.24 pmol; 14.5 µg), the packaging plasmid psPAX2 (1.77 pmol; 11.63 µg), and the envelope plasmid pMD2.G (0.98 pmol; 3.51 µg) in Opti-MEM (Thermo Fisher Scientific, Waltham, MA, USA) to a total volume of 450 µL. For transfection, a polyethylenimine (PEI) solution (1 µg/µL; Polysciences, Hirschberg, Germany) was diluted in 360 µL of Opti-MEM to achieve a PEI:DNA ratio of 3:1. The DNA solution was added to the PEI solution, mixed thoroughly, and incubated at room temperature for 20 min.

The transfection mixture was then added dropwise to the Lenti-X 293T cell culture while gently tilting the flask to ensure even distribution. After 4 h of incubation, the transfection medium was replaced with 15 mL of fresh astrocyte culture medium. The virus-containing supernatant was harvested at 48 and 72 h post-transfection, filtered through a 0.45 µm filter, and pelleted by ultracentrifugation at 25,300× *g* for 2 h at 4 °C. The resulting viral pellet was gently resuspended in PBS, aliquoted, and stored at −80 °C until use.

For quantification, the viral titers were determined using a qPCR Lentivirus Titration LV900 Kit (abm, Richmond, BC, Canada) following the manufacturer’s instructions.

### 2.4. Multiplicity of Infection Testing

To determine the optimal multiplicity of infection (MOI), we tested the transduction efficiency of the GFAP-BFP-T2A-iCre vector in primary rat cortical astrocytes. Primary astrocytes were seeded in poly-D-lysine-coated 24-well plates at a density of 10,000 cells per well in 0.5 mL of astrocyte culture medium. After approximately 24 h, the medium was replaced with 250 µL of astrocyte culture medium containing TransDux (diluted 1:200 to a final concentration of 1×; SBI System Biosciences, Palo Alto, CA, USA). After a 30 min incubation, lentiviral particles were added to achieve final MOIs of 0, 8, 16, 32, 64, 128, and 256.

Twenty-four hours after transduction, the medium was replaced with serum-free astrocyte culture medium (see Table 1). At 72 h post-transduction, cells were washed thoroughly and fixed with 4% paraformaldehyde (PFA). Cell nuclei were stained with RedDot (1:1000 dilution; Biotium, Fremont, CA, USA), and the HA-tag was visualized using the appropriate antibody (see Table 2) following the immunocytochemistry protocol described below. Transduction efficiency was determined by calculating the percentage of BFP+/RedDot+ cells relative to the total number of RedDot+ nuclei in three randomly chosen fields of view.

The optimal MOI for subsequent reprogramming experiments was determined based on these measurements.

### 2.5. Reprogramming Experiments and Tetracycline Induction

For the conversion of astroglia to NPCs or neurons, unless otherwise specified, all cultures were maintained at 37 °C with 5% CO_2_ and 0.5 mL of medium per well throughout the experiments. The specific media formulations used are listed in Table 1, and the medium change schedule is described below.

Primary astrocytes (P0) were plated in poly-D-lysine-coated 24-well plates at a density of 50,000 cells per well [29,30]. On the following day (day 0), the culture medium was replaced with 0.25 mL of astrocyte culture medium containing TransDux (diluted 1:200 to a final concentration of 1×; SBI System Biosciences, Palo Alto, CA, USA). After a 30 min incubation, lentiviral vectors (thawed on ice for 30 min prior to use) were added at an optimal MOI of 32.

When four different vectors—Tet-O-FUW-OSK, FUW-M2rtTA, pGFAP-BFP-T2A-iCre, and either the pNestin-FLEX-EmGFP or pSyn1-FLEX-mScarlet-V5 reporter—were co-transduced, an MOI of 8 was used for each vector to achieve balanced co-transduction.

The following day (day 1, 24 h after lentiviral transduction), the medium was replaced with freshly prepared, pre-warmed (37 °C), and CO_2_-equilibrated serum-free astrocyte culture medium. Forty-eight hours post-transduction (day 2), the medium was changed to neural stem cell (NSC) medium (see Table 1), and expression of Oct4, Sox2, and Klf4 was induced by adding tetracycline (100 ng/mL; Sigma-Aldrich, St. Louis, MO, USA) [30]. Tetracycline-containing NSC medium was refreshed on days 3 and 5 post-transduction.

**Figure 1:**
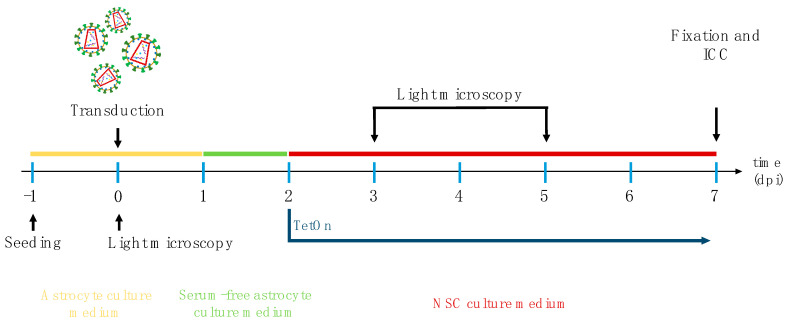
Graphical representation of an astroglia-to-neuron conversion experiment (dpi = days post-infection; ICC = immunocytochemistry; NSC = neural stem cell).

Cell morphology was documented by light microscopy on days 3 and 5 post-transduction. Finally, cells were fixed on day 7 using 4% PFA at room temperature for 20 min (see also Figure 1). Immunocytochemical staining of the fixed cultures was performed according to the protocol described below.

### 2.6. Immunocytochemistry and Imaging

Cells in 24-well plates were fixed with 4% paraformaldehyde (PFA) at room temperature for 20 min. After fixation, they were permeabilized and blocked simultaneously for 10 min at room temperature in a solution containing 0.3% Triton X-100 in blocking solution (PBS with 0.1% Tween-20 and 1% bovine serum albumin (BSA) (Sigma-Aldrich, St. Louis, MO, USA)).

Following permeabilization and blocking, cells were incubated overnight at 4 °C with primary antibodies diluted in blocking solution without Triton X-100 (see Table 2). The next day, cells were washed three times with PBS and incubated for 1 h at room temperature with Alexa Fluor-conjugated secondary antibodies: goat anti-guinea pig (488 nm), donkey anti-mouse (568 nm), and donkey anti-rabbit (647 nm), each at a dilution of 1:500 (all from Abcam, Cambridge, UK), prepared in blocking solution without Triton X-100.

Finally, cell nuclei were stained for 30 min with either DAPI (1:10,000; Sigma-Aldrich, St. Louis, MO, USA) or RedDot (1:1000; Biotium, Fremont, CA, USA). Wells were washed with PBS and then filled with PBS for storage or further analysis.

**Table 2:** Primary antibodies used in the study.

Antibody	Target	Dilution	Species	Company
HA-tag	HA-tag	1:250	Rabbit	Cell Signaling (Danvers, MA, USA)
GFP	GFP	1:400	Sheep	Bio-Rad (Hercules, CA, USA)
GFAP	Astroglia	1:1000	Mouse	Millipore (Sigma-Aldrich, St. Louis, MO, USA)
GLAST	Astroglia	1:250	Guinea pig	Millipore (Sigma-Aldrich, St. Louis, MO, USA)
Sox9	Astroglia	1:1500	Rabbit	Abcam (Cambridge, UK)
S100b	Astroglia	1:100	Rabbit	Sigma-Aldrich (St. Louis, MO, USA)
Iba-1	Microglia	1:500	Rabbit	Invitrogen (Thermo Fisher Scientific, Waltham, MA, USA)
NeuN	Neurons	1:500 1:100	Rabbit Mouse	Millipore (Sigma-Aldrich, St. Louis, MO, USA) Millipore (Sigma-Aldrich, St. Louis, MO, USA)
Syn11	Neurons	1:200	Rabbit	Invitrogen(Thermo Fisher Scientific, Waltham, MA, USA)
β-Tubulin III	Neurons	1:100	Mouse	Sigma Aldrich St. Louis, MO, USA)
Nestin	NPCs	1:200 1:250	Mouse Rabbit Rabbit	Millipore Novus (Sigma-Aldrich, St. Louis, MO, USA) Abcam (Cambridge, UK)
DCX	NPCs	1:200	Rabbit	Invitrogen (Thermo Fisher Scientific, Waltham, MA, USA)
Olig2	Oligodendrocytes	1:200	Rabbit	Millipore (Sigma-Aldrich, St. Louis, MO, USA)

### 2.7. Image Analysis and Cell Quantification

Three tile scans (each measuring 6544 × 339.8 µm^2^) were acquired from each well at random locations using an LSM700 confocal laser scanning microscope (Carl Zeiss GmbH, Oberkochen, Germany) in 16-bit format.

Confocal images from the primary astrocyte cultures were subjected to semi-automatic cell quantification of marker expression using the ImageJ/Fiji software package version 1.53k (ImageJ2, U.S. National Institutes of Health, Bethesda, MD, USA). Briefly, images were split into individual fluorescence channels, and background noise was reduced using a Gaussian filter. The “Image Calculator” function was applied to enhance contrast. Each channel was then converted into a binary image, with threshold values individually adjusted for each antibody using the “Threshold” settings (“default,” “B&W,” “dark background”). Regions of interest (ROIs) were drawn manually to exclude large artifacts or autofluorescent well edges. The “Analyze Particles” function was used to count threshold-exceeding structures within the ROIs, and the “Wand Tracing Tool” and “Measure” functions were employed to refine size thresholds and exclude artifacts. Colocalization of two channels was assessed for all possible combinations to exclude non-overlapping structures and to confirm co-expression of markers.

For cell quantification in conversion experiments, confocal images were analyzed using the open-source software QuPath (version 0.4.3) [31]. In short, an ROI encompassing the entire image of a well (excluding autofluorescent well edges) was annotated. Cells were counted using the “Cell Detection” analysis, with nuclear staining displayed as a grayscale image to set the appropriate intensity threshold for detection. A Gaussian filter (σ = 2) was applied to reduce background noise, and nuclear size thresholds were refined using the “Wand Annotation” tool to exclude artifacts. For each antibody, a “Single Measurement Classifier” was created using “Live Preview” and sequentially applied to the ROI to quantify marker expression.

### 2.8. Statistical Analysis

All results are presented as mean ± standard error of the mean (SEM) of at least three different wells in three independent experiments unless stated otherwise.

For statistical comparisons among three or more groups, one-way analysis of variance (ANOVA) followed by post hoc Tukey’s HSD tests was applied. If standard deviations differed significantly, Welch’s ANOVA was used, with multiple comparisons adjusted by the Dunnett T3 correction.

For comparisons between two groups, unpaired *t*-tests were performed. If variances differed significantly, Welch’s correction was applied. In cases of non-normally distributed data, the Mann–Whitney test was used instead of parametric tests.

All statistical tests were conducted at a predefined significance level of α = 0.05. Analyses were performed using the Prism software (version 10; GraphPad, Boston, MA, USA).

## 3. Results

### 3.1. Design of the Reporter System

To establish a robust tool for monitoring the transition of astrocytes towards neuronal NPCs or neurons, we designed a GFAP-dependent neuronal reporter system. This system was intended to provide an in vitro platform to visualize potential reprogramming events, laying the groundwork for future in vivo applications.

**Figure 2:**
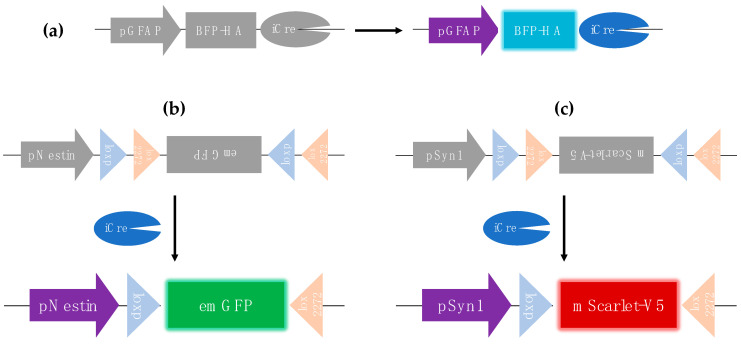
(**a**) Astrocytes transduced by the pGFAP-BFP-HA-iCre lentivirus express BFP, an HA-tag, and iCre recombinase. (**b**,**c**) Upon iCre expression, the reversely oriented double lox sequences in the Nestin-EmGFP and Synapsin1-mScarlet reporter constructs are inverted, reorienting the cDNA of the fluorescent reporters. Reporter expression occurs only when the respective neuronal promoters—Nestin (for NPC-like states) or Synapsin1 (for more mature neuronal features)—are activated, reflecting potential reprogramming events from astrocytes to neuronal precursor cells or neurons.

To facilitate monitoring of reprogramming outcomes over time, the reporter system comprises three distinct constructs that collectively enable the detection of a cellular shift from GFAP expression to either Synapsin-1 or Nestin expression. Specifically, the following is conducted:

○ The Nestin promoter drives a double-floxed, reverse-oriented EmGFP cassette (Figure 2b), enabling detection of intermediate NPC-like states.
○ The Synapsin-1 promoter drives a double-floxed, reverse-oriented mScarlet cassette (Figure 2c), indicating more mature neuronal features.

In the initial lentiviral transduction, GFAP-driven BFP-HA-iCre is expressed in astrocytes (Figure 2a), mediating the inversion of the reverse-oriented reporter cassette. Notably, the other two reporter fluorescent proteins are not expressed in GFAP-positive astrocytes themselves. Their expression occurs only if the respective neuronal promoters (Nestin or Synapsin-1) are activated, suggesting progression towards NPC or neuronal-like phenotypes.

This system thus provides a technical framework to track astrocyte lineage transitions under OSK-mediated reprogramming in vitro.

### 3.2. Validation of Bona Fide Primary Astrocytes

**Figure 3:**
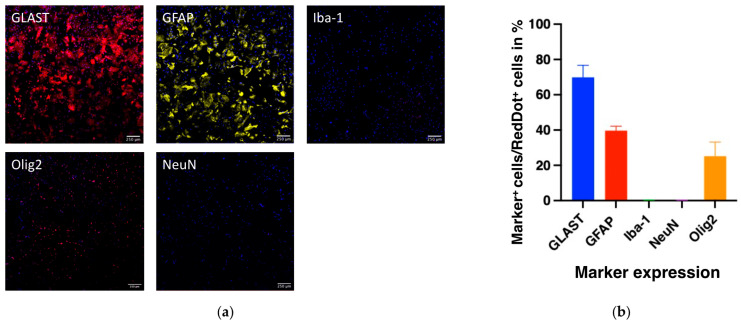
Characterization of the astroglia-enriched primary cell culture derived from rat pup cortices. (**a**) Immunocytochemistry staining with primary antibodies for GLAST (*red*), GFAP (*yellow*), Iba-1 (*red*), Olig2 (*red*), and NeuN (*red*) co-stained with DAPI (*blue*). (**b**) Histogram showing the percental marker expression in the primary astrocyte cell culture.

To estimate the purity of our astrocyte-enriched primary cultures, we analyzed the expression of cell-type markers for astrocytes (GFAP, GLAST), neurons (NeuN), microglia (Iba-1), and oligodendrocyte lineage cells (Olig2) (Figure 3a).

Consistently, the highest marker expression was observed for the astrocytic markers: GLAST: 69.88 ± 6.681% and GFAP: 39.61 ± 2.556%. As expected, the presence of microglia and neuronal cells was minimal (Iba-1: 0.22 ± 0.044%, NeuN: 0.23 ± 0.069%; Figure 3b). These results likely reflect the selective removal of microglia through gentle tapping during medium changes and the use of an astrocyte-optimized medium, which suppresses neuronal cell growth.

**Figure 4:**
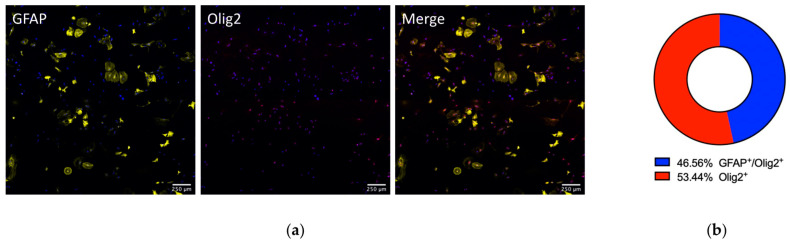
Characterization of the Olig2^+^ population in the astroglia-enriched primary cultures. (**a**) Co-staining of the cells with primary antibodies for GFAP (*yellow*), Olig2 (*red*), and DAPI (*blue*). (**b**) Graph indicating the percentage of the GFAP^+^/Olig2^+^ astrocytes versus only Olig2-only positive cells within the cultures.

Interestingly, we observed a relatively high proportion of Olig2-positive cells (25.19 ± 8.045%) in these cultures, prompting a closer examination of this subpopulation. Co-localization analysis revealed that approximately 46.56% of the Olig2-positive cells co-expressed GFAP, indicating they were Olig2-expressing astrocytes—a population known to exist in the brains and spinal cords of postnatal rats [32,33] (Figure 4). From this, we inferred that the percentage of bona fide oligodendrocytes (Olig2+/GFAP–) in our cultures was about 13.46%. Consequently, the estimated proportion of bona fide astrocytes in our primary cultures was approximately 86.54%.

Taken together, these results confirm that our primary astrocyte cultures—despite some heterogeneity—were predominantly composed of astrocytes, supporting their suitability for testing our reporter system in astrocyte-to-neuron conversion experiments. This phenotypic profile provides a baseline against which to interpret potential lineage shifts during conversion experiments.

### 3.3. Determining the Optimal Multiplicity of Infection

To prepare for the conversion experiments, we first determined the optimal MOI for the lentiviral vectors. We used the pGFAP-BFP-T2A-iCre vector for this purpose because its transduction efficiency can be directly assessed by counting BFP-positive fluorescent cells under a confocal microscope. As a control, wells with the same primary astrocyte culture but without viral vectors were included, and transduction efficiency was calculated as the percentage of BFP^+^/RedDot^+^ cells out of all RedDot^+^ nuclei. This served as a proxy for vector delivery efficiency.

**Figure 5:**
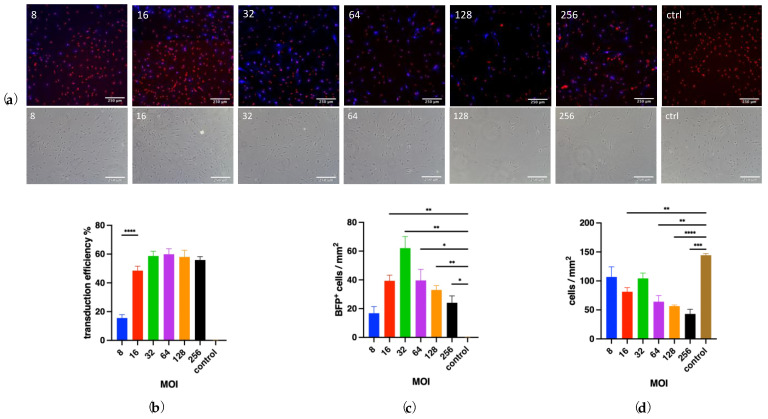
Determination of the optimal multiplicity of infection. (**a**) Cultured astrocytes transduced with pGFAP-BFP-T2A-iCre (MOIs 8, 16, 32, 64, 128, 256) and control wells (no virus). BFP expression (*blue*) was enhanced by immunocytochemistry staining of the HA-tag, while nuclei were labeled with RedDot (*red*). Corresponding bright-field images show cell density and morphology at the different MOIs. (**b**) Histogram displaying transduction efficiency for each MOI, defined as the percentage of BFP^+^/RedDot^+^ cells out of all RedDot^+^ cells; **** *p* < 0.0001. (**c**) Histogram showing the density (cells/mm^2^) of BFP-positive cells across MOIs; * *p* < 0.0332, ** *p* < 0.0021. (**d**) Histogram showing the total cell density (RedDot^+^ cells/mm^2^) as an indicator for cell viability across MOIs; ** *p* < 0.0021, *** *p* < 0.0002, **** *p* < 0.0001.

Morphological analysis of bright-field images revealed no major differences in cell morphology between MOIs 8 and 64. In these conditions, cells grew in dense networks with numerous spatial contacts and displayed round-to-oval nuclei. However, at higher MOIs (128 and 256), cultures appeared less dense (Figure 5a), suggesting increased cytotoxicity due to higher viral loads.

As expected, the control group showed no relevant transduction (0.1280 ± 0.02311%). The transduction efficiency increased progressively with MOI up to 64 (MOI 8: 15.55 ± 2.320%; MOI 16: 48.47 ± 3.167%; MOI 32: 58.70 ± 3.292%; MOI 64: 59.90 ± 3.793%), with the curve flattening from MOI 32 to 64. At higher MOIs (128 and 256), a slight decrease in transduction efficiency was observed (MOI 128: 58.05 ± 4.660%; MOI 256: 55.92 ± 2.291%). Only the transition from MOI 8 to MOI 16 showed a statistically significant increase in transduction rate (*p* < 0.0001), whereas higher MOIs did not differ significantly from each other (Figure 5b).

Comparison of each MOI group to the control group showed significant (MOI 8: *p* = 0.0168) to highly significant (MOI 16–256, *p* < 0.0001) increases in the absolute number of BFP^+^ cells/mm^2^ (Figure 5c).

In addition to morphology and transduction efficiency, total cell counts (RedDot^+^ cells/mm^2^) provided an indirect measure of cell viability and proliferation (Figure 5d). The control group, which was not exposed to viral transduction stress, showed the highest average cell count (144.4 ± 2.861 RedDot^+^ cells/mm^2^). In contrast, increasing MOI generally resulted in decreasing cell density. While MOI 8 exhibited the highest cell count among transduced wells (107.0 ± 17.39 RedDot^+^ cells/mm^2^), the highest MOI tested (MOI 256) had significantly lower cell density (43.03 ± 8.083 RedDot^+^ cells/mm^2^).

Based on these results, we selected MOI 32 as the optimal MOI for subsequent reprogramming experiments because it demonstrated high transduction efficiency while maintaining good cell viability and density.

### 3.4. Conversion of Astrocytes to Neurons or Neuronal Precursors

For the conversion experiments, the astrocyte-enriched primary cultures were transduced with lentiviral vectors containing the reporter system (pGFAP-BFP-T2A-iCre, pNestin-FLEX-EmGFP, or pSyn1-FLEX-mScarlet) and the Tet-On system (FUW-M2rtTA). The experimental group additionally received the vector carrying the transcription factors Oct4, Sox2, and Klf4 (OSK), while the control group was transduced with the same reporters and Tet-On vectors but without the OSK cassette. Following the conversion protocol, cells were fixed at 7 days post-infection (dpi) and analyzed by immunocytochemistry (ICC).

#### 3.4.1. Morphological Changes

**Figure 6:**
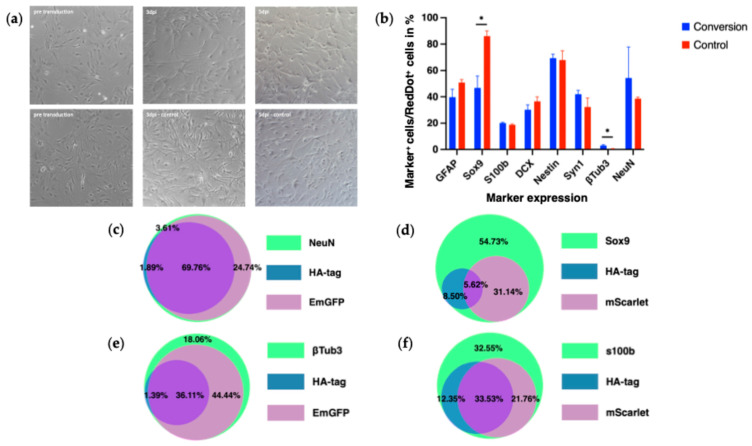
Assessments made to characterize the cells during and after the conversion experiment. (**a**) Bright-field images that show morphological changes observed in the experimental and control group at different time points (prior to transduction, 3 dpi and 5 dpi); (**b**) Histogram showing the percental marker expressions after fixation at 7 dpi; * *p* = 0.02. (**c**) Composition of the subpopulation of NeuN^+^ cells after fixation at 7 dpi [34]. (**d**) Composition of the subpopulation of Sox9^+^ cells after fixation at 7 dpi [34]. (**e**) Composition of the subpopulation of β-Tubulin III^+^ cells after fixation at 7 dpi [34]. (**f**) Composition of the subpopulation of S100b^+^ cells after fixation at 7 dpi [34].

To assess early cellular responses to OSK transduction, bright-field images were acquired at 3 dpi and 5 dpi to observe morphological changes (Figure 1). Initially, cells displayed a typical flat, polygonal morphology with an even distribution across the plates. By day 3 post-infection, a subpopulation of cells exhibited a spindle-shaped morphology with elongated, thin processes and smaller somata, which became more pronounced by 5 dpi (Figure 6a). Importantly, these morphological changes were observed in both the experimental and control groups.

#### 3.4.2. Marker Expression

**Figure 7:**
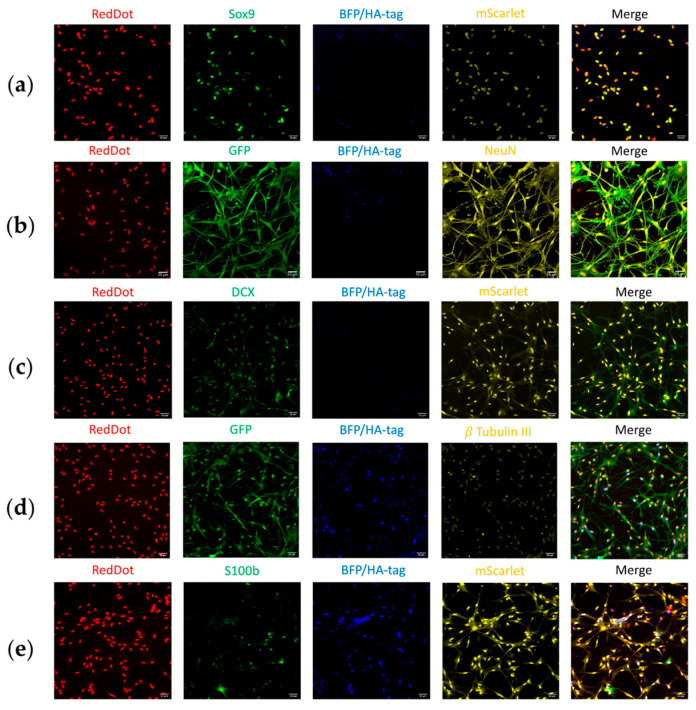
Immunocytochemical stainings of the primary astrocyte cultures in the conversion groups after fixation at 7 dpi. (**a**) Staining for Sox9 (green), the reporter BFP/HA-tag (blue), mScarlet (yellow), and RedDot (red), indicating cells with astrocytical and neuronal features (Merge). (**b**) Staining for NeuN (yellow) with the reporter BFP/HA-tag (blue), EmGFP (green), and RedDot (red), indicating cells with NPC and neuronal features (Merge). (**c**) Staining for DCX (green) with the reporter BFP/HA-tag (blue), mScarlet (yellow), and RedDot (red), indicating cells with NPC and neuronal features (Merge). (**d**) Staining for β-Tubulin III (yellow) with the reporter BFP/HA-tag (blue), EmGFP (green), and RedDot (red), indicating cells with NPC and neuronal features (Merge). (**e**) Staining for S100b (green) with the reporter BFP/HA-tag (blue), mScarlet (yellow), and RedDot (red), indicating cells with astrocytical and neuronal features (Merge).

To characterize cell identity after the conversion experiments, we performed ICC staining for various markers (Figure 7), including astrocytic markers (GFAP, Sox9, S100b), neuronal markers (NeuN, Synapsin-1 (Syn1), β-Tubulin III), and progenitor cell markers (Nestin, DCX).

Astrocytic markers showed moderate expression levels post-conversion: GFAP: 39.7 ± 5.94% and Sox9: 46.7 ± 9.04% of all RedDot-positive cells. These percentages were similar to pre-conversion levels (Figure 3b). S100b was expressed by 20.1 ± 0.645% of cells in the conversion group. In the control group, GFAP expression was slightly higher (50.8 ± 2.28%), and S100b was slightly lower (18.7 ± 0.695%). Only Sox9 showed a significant decrease in the conversion group (86.0 ± 3.94% in control vs. 46.7 ± 9.04% in conversion, *p* = 0.02) (Figure 6b).

Among neuronal markers, Syn1 was expressed in 42.0 ± 2.91% of cells in the conversion group, not significantly different from the control group control (32.3 ± 6.85%, *p* = 0.26). NeuN expression was observed in 60.9 ± 17.5% of conversion cells versus 38.6 ± 1.16% in controls (*p* = 0.33). Notably, the marker for mature neurons, β-Tubulin III, was expressed in only a few cells (2.99 ± 0.674% in conversion), but this was significantly higher than in controls (0.463 ± 0.240%, *p* = 0.02; Figure 6b).

Stem cell markers showed no significant differences: DCX was expressed in 30.2 ± 3.68% of conversion cells (control: 36.5 ± 3.45%, *p* = 0.28). Nestin was highly expressed in both groups (conversion: 69.3 ± 3.05%, control: 68.0 ± 6.98%, *p* = 0.86; (Figure 6b). These data served as the foundation for interpreting reporter activation in the following analyses.

#### 3.4.3. Reporter System and Corresponding Marker Expression

**Figure 8:**
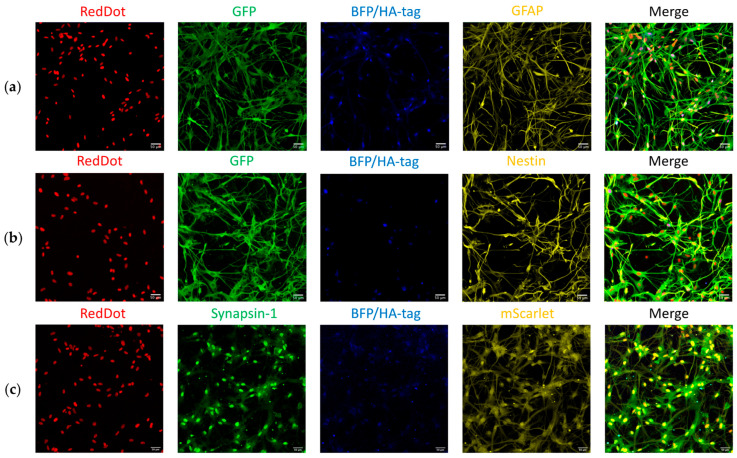
Immunocytochemical staining of primary astrocyte cultures in the conversion groups after fixation at 7dpi. (**a**) Staining for GFAP (*yellow*) with the reporter BFP/HA-tag (*blue*), EmGFP (*green*), and RedDot (*red*), highlighting astrocytic and NPC-like features (merged image). (**b**) Staining for Nestin (*yellow*) with the reporter BFP/HA-tag (*blue*), EmGFP (*green*), and RedDot (*red*), illustrating NPC-like characteristics (merged). (**c**) Staining for Synapsin-1 (*green*) with the reporter BFP/HA-tag (*blue*), mScarlet (*yellow*), and RedDot (*red*), indicating cells with neuronal features (merged).

The functionality of the reporter system was assessed by visualizing the expression of the reporter fluorescent proteins (BFP/HA-tag, mScarlet, and EmGFP) (Figure 8). BFP/HA-tag marked GFAP-positive cells, mScarlet marked Synapsin-1-positive cells, and EmGFP marked Nestin-positive cells, and their quantification enabled assessment of promoter activity post-conversion.

**Figure 9:**
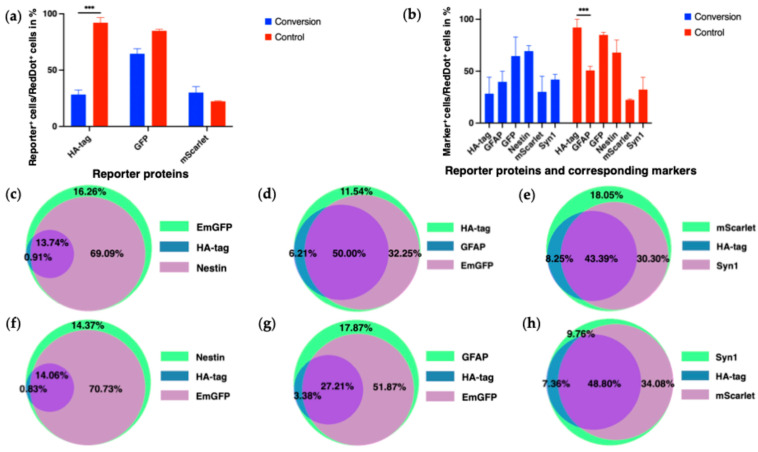
Characterization of the reporter system expression and its relationship with specific marker expression after conversion experiments. (**a**) Histogram showing the percentage of cells expressing the reporter proteins HA-tag, EmGFP, and mScarlet in conversion and control groups after fixation at 7 dpi; *** *p* < 0.001. (**b**) Histogram comparing the percentage of reporter expression with their corresponding endogenous markers GFAP, Nestin, and Synapsin-1 (Syn1) at 7 dpi; *** *p* < 0.001. (**c**–**h**) Subpopulation analyses showing the overlap of marker and reporter expression [34]: (**c**) EmGFP^+^ cells, (**d**) HA-tag^+^ cells, (**e**) mScarlet^+^ cells, (**f**) Nestin^+^ cells, (**g**) GFAP^+^ cells, (**h**) Synapsin-1^+^ cells at 7 dpi.

Quantification of reporter fluorescence showed that only BFP/HA-tag expression differed significantly between groups (conversion: 28.4 ± 3.97%, control: 92.1 ± 4.61%, *p* < 0.001). Expression of mScarlet (conversion: 30.1 ± 5.33%, control: 22.3 ± 0.470%, *p* = 0.19) and EmGFP (conversion: 64.5 ± 4.46%, control: 84.9 ± 1.44%, *p* = 0.08) showed no significant differences. These trends reflect both biological processes and potential differences in reporter stability or silencing (Figure 9a).

We further analyzed the relationship between reporter expression and their corresponding markers (GFAP, Syn1, Nestin) (Figure 9b). Except for the BFP/HA-tag and GFAP in the control group (*p* < 0.001), no significant differences were observed. In the control group, BFP/HA-tag expression (92.1 ± 2.91%) was significantly higher than GFAP expression (50.8 ± 2.28%).

Co-localization analyses confirmed substantial overlap between reporter expression and their target markers:

○ EmGFP/Nestin: 82.8% (69.09% EmGFP/Nestin + 13.74% EmGFP/Nestin/HA-tag) of EmGFP-positive cells stained for Nestin; 84.8% (70.73% Nestin/EmGFP + 14.06% Nestin/EmGFP/HA-tag) of Nestin-positive cells expressed EmGFP (Figure 9c,f).
○ mScarlet/Syn1: 82.9% (34.08% Syn1/mScarlet + 48.80% Syn1/mScarlet/HA-tag) of Syn1-positive cells expressed mScarlet; 73.7% (30.30% mScarlet/Syn1 + 43.39% mScarlet/Syn1/HA-tag) of mScarlet-positive cells stained for Syn1 (Figure 9e,h).
○ BFP/HA-tag/GFAP: lower co-localization—56.2% (6.21% HA-tag/GFAP + 50.00% HA-tag/GFAP/EmGFP) of HA-tag-positive cells stained for GFAP; only 30.6% (3.38% GFAP/HA-tag + 27.21% GFAP/HA-tag/EmGFP) of GFAP-positive cells expressed the HA-tag (Figure 9d,g).

We also assessed cross-colocalization of the HA-tag with mScarlet and EmGFP. Interestingly, while the reporter system design (Figure 2) would predict high co-expression, only 51.6% of mScarlet-positive cells and 14.7% of EmGFP-positive cells expressed the HA-tag.

## 4. Discussion

In this study, we developed an in vitro system to explore the potential conversion of astrocytes to neuronal precursor cells or neurons using a primary rat pup cortical astrocyte culture, OSK transcription factor delivery via lentiviral vectors, and a tailored reporter system to monitor lineage marker transitions. Our data demonstrate that while OSK overexpression influenced the expression of several neuronal and astrocytic markers, the observed effects were modest and likely shaped by a complex interplay between the in vitro microenvironment, growth factors, and the transcription factors themselves. These findings highlight both the promise and the current limitations of this approach, laying a technical foundation for future investigations into astrocyte reprogramming, particularly the application and refinement of viral reporter tools for lineage tracing in both in vitro and in vivo settings.

### 4.1. Reporter System

#### 4.1.1. Evaluation of Reporter and Endogenous Marker Co-Localization

The designed reporter system allowed for initial in vitro validation of the astrocyte reprogramming approach. Notably, the reporters mScarlet and EmGFP exhibited high levels of co-localization with their respective endogenous markers, Synapsin-1 and Nestin, aligning with their intended roles as indicators of neuronal and progenitor-like features. In contrast, the GFAP-dependent reporter (BFP/HA-tag) did not fully overlap with GFAP expression (Figure 9d,g). This discrepancy likely reflects known position effects or epigenetic-mediated silencing following random genomic integration of the lentiviral vectors [21,35]. While transgenic lineage tracing using Cre-dependent reporters in GFAP-Cre or Aldh1l1-CreERT2 mice would offer more definitive proof of astrocyte origin, our lentiviral system provides a flexible and translationally oriented platform, especially suited for evaluating viral vector strategies in future therapeutic studies [36].

Additionally, the truncated pGFAP promoter we employed—lacking the silencer sequence—may have broadened expression beyond the endogenous GFAP pattern. We observed that only 56.2% of HA-tag-positive cells were also GFAP-positive, consistent with our MOI tests (Figure 5) and marker analysis in primary cultures (Figure 3). Moreover, in the GFAP-positive subpopulation of the conversion experiments, only 30.6% of cells expressed the HA-tag. This may be due to incomplete transduction, conversion-associated downregulation of the pGFAP promoter, or the persistence of the GFAP protein itself, which has a long half-life of about 8 days and thus remains detectable via immunofluorescence despite reporter silencing [37].

#### 4.1.2. Interpretation of Reporter–Marker Co-Localizations

We defined a cell as “converted” if it expressed both the HA-tag reporter and either the mScarlet or EmGFP reporter. In these cells, the presence of the HA-tag indicated active GFAP promoter expression prior to the conversion, while the activation of the Nestin or Synapsin-1 promoters and the expression of EmGFP or mScarlet suggested progression towards neuronal precursor or neuronal-like states.

For example, within the Synapsin-1-positive subpopulation, the following distinctions were observed:

1. HA-tag^+^, mScarlet^+^, Synapsin-1^+^ cells likely represent converted cells retaining some astrocytic characteristics while acquiring neuronal features.
2. HA-tag^+^, Synapsin-1^+^ cells did not co-express mScarlet, suggesting partial reporter uptake or incomplete conversion.
3. mScarlet^+^, Synapsin-1^+^ cells lacked HA-tag expression, indicating potential conversion with diminished GFAP promoter activity.
4. Synapsin-1^+^ cells alone lacked reporter expression, suggesting incomplete co-transduction of the reporter system.

Overall, 51.6% of mScarlet-expressing cells and 14.7% of EmGFP-expressing cells still expressed the HA-tag reporter, indicating partial retention of GFAP promoter activity in these reprogramming transitions. These findings are consistent with observations by Masserdotti et al. [29] who reported similar co-expression patterns in cells undergoing direct conversion from astrocytic to neuronal fates.

#### 4.1.3. Relevance of EmGFP/Nestin as a Reporter for Cell Conversion

Among the reporter constructs, EmGFP driven by the Nestin promoter showed high expression within the GFAP-positive subpopulation, with approximately 79% of GFAP-positive cells also expressing Nestin. This observation is consistent with previous studies showing co-expression of GFAP and Nestin in cortical astrocytes of postnatal rodents [19,38,39,40].

These results suggest that while Nestin is a reliable marker of progenitor-like states, its pre-existing co-expression with GFAP complicates the interpretation of conversion events in neonatal astrocyte cultures. Consequently, EmGFP/Nestin as a reporter for conversion may not reliably distinguish newly converted neuronal precursor cells from already-Nestin-expressing astrocytes in these early postnatal cultures.

Nevertheless, since EmGFP expression in this reporter system is conditional on prior GFAP promoter activation (Figure 2), it could still be valuable for future in vivo studies or experiments using older astrocyte populations, where GFAP and Nestin co-expression is less prominent. Additionally, the documented presence of GFAP+/Nestin+ cells in traumatic brain injury (TBI) models [41,42] highlights a potential application for this reporter system in in vivo injury contexts. However, the pre-existing co-expression of these markers in neonatal cultures suggests caution when interpreting EmGFP as a conversion indicator in vitro.

### 4.2. Conversion

#### 4.2.1. Trends in Marker Expression

Our analysis of marker expression in the conversion experiments focused on comparing the experimental group (transduced with OSK) and the control group (lacking OSK but otherwise identically treated). Overall, only a few statistically significant differences were observed. However, trends emerged that suggest a subtle shift in cell identity: astrocytic marker expression was slightly reduced in the conversion groups, whereas neuronal markers tended to be more prevalent.

Specifically, while markers such as GFAP and S100b showed only minor decreases in the conversion group compared to controls, Sox9 expression was significantly lower in the OSK-treated cultures (*p* = 0.02). Neuronal markers, including NeuN, Synapsin-1, and β-Tubulin III, exhibited increased expression in the conversion group, though these differences did not reach statistical significance, except for β-Tubulin III, which was significantly upregulated (*p* = 0.02). These observations suggest an initial, albeit modest, shift towards a neuronal-like phenotype.

Importantly, we did not assess changes in marker fluorescence intensity at the single-cell level, meaning that possible increases or decreases in marker expression within individual cells were not captured. Additionally, the co-localization of astrocytic, neuronal, and progenitor markers in many cells suggests a transitional state rather than complete lineage conversion. Since this study only examined the early phase of reprogramming (7 dpi), further experiments with longer culture periods and differentiation-promoting conditions are needed to clarify the eventual fate of these partially reprogrammed cells. Importantly, the observation of cells in transitional states—expressing both astrocytic and neuronal markers—may itself be a valuable model to study early reprogramming dynamics, including epigenetic remodeling, promoter silencing, and partial lineage identity. This further supports the utility of the reporter system as a live tool to dissect these processes over time.

#### 4.2.2. How Effective Was the Reprogramming?

The modest and mostly non-significant differences in marker expression between the conversion and control groups suggest that only a limited fraction of astrocytes underwent reprogramming under our experimental conditions. Several factors likely contributed to this outcome:

First, while the OSK transcription factor trio is well known for reprogramming somatic cells to induced pluripotent stem cells (iPSCs) [7], the culture conditions in our experiments (astrocyte medium and NSC medium) were tailored more towards neuronal lineage induction [18,43]. OSK was selected due to its established role in reprogramming somatic cells toward pluripotency and emerging evidence suggesting that these factors, individually or in combination, may induce neural traits under certain environmental conditions [7,18]. This context aligns with the observed trends and the significant reduction in Sox9 and increase in β-Tubulin III expression. However, it also raises the possibility that growth factors present in the NSC medium—rather than OSK alone—may have contributed to the observed marker shifts in both the experimental and control groups. Wang et al. [36] highlight the need for caution in interpreting reporter^+^/neuronal-marker^+^ cells as evidence of true astrocyte conversion, as some cells might be endogenous neurons rather than reprogrammed astrocytes. Similarly, Zeng et al. [44] showed that neonatal astrocytes can spontaneously adopt neuronal characteristics in insulin-containing media—paralleling the insulin used in our G5 supplement. Imura et al. [19] also observed spontaneous neuronal marker expression in serum-free astrocyte cultures, underscoring the possibility that growth factors and medium composition play crucial roles independent of OSK overexpression.

Second, in vitro reprogramming studies typically use fluorescent reporters co-expressed with the reprogramming factors to label cells transduced with the genes of interest (GOI) [29,45,46]. In our system, the GOI and reporters were in separate vectors, making it impossible to directly correlate neuronal marker expression with OSK transduction at the single-cell level. This separation complicates comparisons with other reprogramming studies that use integrated reporter/GOI constructs.

Third, the timeframe of our experiments (7 dpi) may have been too short to capture complete conversion events. Previous studies have demonstrated that neuronal reprogramming, especially with different transcription factors, can require extended culture periods of several weeks to achieve full neuronal differentiation and functional maturity [47]. Future experiments with longer culture durations and electrophysiological assessments will be critical to determine the true reprogramming potential of OSK in this system.

### 4.3. Translation to In Vivo Models of TBI

Several studies have explored similar reprogramming approaches in vivo, often using different transcription factors or vectors and various cell types and species, including mice and human astrocytes [9,48,49,50]. For instance, Lu et al. [51] delivered OSK to mouse retinal ganglion cells using adeno-associated viruses (AAVs), highlighting the potential of OSK to influence neuronal phenotypes in the adult nervous system. Interestingly, many in vivo studies use single factors—particularly Oct4—as potent drivers of cellular reprogramming [9,18].

In the context of neurotrauma, only a few studies have directly investigated reprogramming approaches in traumatic brain injury (TBI) or cortical injury models [9,18], as well as in spinal cord injury (SCI) [52]. These studies typically rely on transcription factor overexpression to modify cell fate, and while some groups have used lentiviral vectors [52], AAV vectors are more common due to their non-integrating nature and lower immunogenicity [53]. Most in vivo TBI studies have employed stab-injury models in rodent cortices [54,55,56], with primary assessments focusing on cellular and molecular endpoints.

To our knowledge, this is the first study to apply the OSK transcription factor trio to primary rat cortical astrocytes using lentiviral delivery. The reporter system and methodological framework we developed here could inform future experiments that aim to track and evaluate in vivo conversion processes. While further work is needed to determine functional outcomes, the use of OSK in this context lays the groundwork for dissecting transcription factor interactions and lineage transitions in a neurotrauma setting. However, for any future in vivo translation, it will be important to use more clinically relevant TBI models, such as the controlled cortical impact (CCI) model [57], and to incorporate functional recovery assessments (e.g., gait analysis) to better understand the therapeutic potential of reprogrammed cells.

## 5. Conclusions

This study presents the development of an in vitro system to explore the reprogramming of primary rat cortical astrocytes using the transcription factors Oct4, Sox2, and Klf4 (OSK) delivered via lentiviral vectors. By combining this approach with a tailored reporter system, we were able to monitor transitions in marker expression that suggest early shifts towards neuronal or progenitor-like phenotypes. Our data indicate that while OSK overexpression induced modest changes in astrocytic and neuronal marker expression, the conversion process remained incomplete, with many cells in a transitional state co-expressing astrocytic, neuronal, and progenitor markers.

These findings highlight the technical feasibility of using a GFAP-dependent reporter system to track potential lineage transitions in vitro. However, they also underscore the complexity of astrocyte reprogramming and the significant challenges that remain. The observed trends in marker expression may have been influenced by the culture conditions themselves, including growth factors present in the media, and the lack of direct linkage between the OSK cassette and the reporters complicates definitive attribution of marker shifts to OSK-mediated conversion.

Future studies should aim to refine the in vitro protocol by optimizing vector delivery, adjusting transcription factor expression levels, and extending the culture duration to enable more complete reprogramming. Additionally, the microenvironmental influences on astrocyte fate decisions—particularly in in vivo injury contexts—deserve further investigation. Ultimately, the methods and observations presented here provide a technical platform for evaluating astrocyte plasticity and establishing in vivo experiments to assess the functional and therapeutic potential of reprogrammed astrocytes in clinically relevant models.

## Figures and Tables

**Table 1:** Cell culture media used in the experiments.

Cell Culture Media	
Astrocyte culture medium	DMEM + 4.5 g/L glucose (Gibco, Carlsbad, CA, USA) 10% FBS (Gibco, Carlsbad, CA, USA) 1% penicillin/streptomycin (Thermo Fisher Scientific, Waltham, MA, USA)
Serum-free astrocyte medium	DMEM/F12 (Gibco, Carlsbad, CA, USA) 1% G5 (Gibco, Carlsbad, CA, USA) 1% penicillin/streptomycin (Thermo Fisher Scientific, Waltham, MA, USA)
NSC medium [18]	DMEM/F12 (Gibco, Carlsbad, CA, USA) 1% N2 (Gibco, Carlsbad, CA, USA) 25 ng/mL basic fibroblast growth factor (Sigma-Aldrich, St. Louis, MO, USA) 20 ng/mL epidermal growth factor (Sigma-Aldrich, St. Louis, MO, USA) 1% penicillin/streptomycin (Thermo Fisher Scientific, Waltham, MA, USA)

## Data Availability

The raw data supporting the conclusions of this article will be made available by the authors on reasonable request.
